# Beyond Adoption: A New Framework for Theorizing and Evaluating Nonadoption, Abandonment, and Challenges to the Scale-Up, Spread, and Sustainability of Health and Care Technologies

**DOI:** 10.2196/jmir.8775

**Published:** 2017-11-01

**Authors:** Trisha Greenhalgh, Joseph Wherton, Chrysanthi Papoutsi, Jennifer Lynch, Gemma Hughes, Christine A'Court, Susan Hinder, Nick Fahy, Rob Procter, Sara Shaw

**Affiliations:** ^1^ Department of Primary Care Health Sciences University of Oxford Oxford United Kingdom; ^2^ School of Health and Social Work University of Hertfordshire Hatfield United Kingdom; ^3^ RAFT Research and Consulting Ltd Clitheroe, Lancs United Kingdom; ^4^ Department of Computer Science University of Warwick Coventry United Kingdom

**Keywords:** diffusion of innovation, scale-up, program sustainability, implementation, complexity of innovations, business planning, NASSS framework, nonadoption, abandonment, scale-up, spread, sustainability framework, innovation adoption

## Abstract

**Background:**

Many promising technological innovations in health and social care are characterized by nonadoption or abandonment by individuals or by failed attempts to scale up locally, spread distantly, or sustain the innovation long term at the organization or system level.

**Objective:**

Our objective was to produce an evidence-based, theory-informed, and pragmatic framework to help predict and evaluate the success of a technology-supported health or social care program.

**Methods:**

The study had 2 parallel components: (1) secondary research (hermeneutic systematic review) to identify key domains, and (2) empirical case studies of technology implementation to explore, test, and refine these domains. We studied 6 technology-supported programs—video outpatient consultations, global positioning system tracking for cognitive impairment, pendant alarm services, remote biomarker monitoring for heart failure, care organizing software, and integrated case management via data sharing—using longitudinal ethnography and action research for up to 3 years across more than 20 organizations. Data were collected at micro level (individual technology users), meso level (organizational processes and systems), and macro level (national policy and wider context). Analysis and synthesis was aided by sociotechnically informed theories of individual, organizational, and system change. The draft framework was shared with colleagues who were introducing or evaluating other technology-supported health or care programs and refined in response to feedback.

**Results:**

The literature review identified 28 previous technology implementation frameworks, of which 14 had taken a dynamic systems approach (including 2 integrative reviews of previous work). Our empirical dataset consisted of over 400 hours of ethnographic observation, 165 semistructured interviews, and 200 documents. The final nonadoption, abandonment, scale-up, spread, and sustainability (NASSS) framework included questions in 7 domains: the condition or illness, the technology, the value proposition, the adopter system (comprising professional staff, patient, and lay caregivers), the organization(s), the wider (institutional and societal) context, and the interaction and mutual adaptation between all these domains over time. Our empirical case studies raised a variety of challenges across all 7 domains, each classified as simple (straightforward, predictable, few components), complicated (multiple interacting components or issues), or complex (dynamic, unpredictable, not easily disaggregated into constituent components). Programs characterized by complicatedness proved difficult but not impossible to implement. Those characterized by complexity in multiple NASSS domains rarely, if ever, became mainstreamed. The framework showed promise when applied (both prospectively and retrospectively) to other programs.

**Conclusions:**

Subject to further empirical testing, NASSS could be applied across a range of technological innovations in health and social care. It has several potential uses: (1) to inform the design of a new technology; (2) to identify technological solutions that (perhaps despite policy or industry enthusiasm) have a limited chance of achieving large-scale, sustained adoption; (3) to plan the implementation, scale-up, or rollout of a technology program; and (4) to explain and learn from program failures.

## Background

In 2004 and 2005, Greenhalgh et al published a multilevel framework for studying diffusion of innovations in health care, based on a cross-disciplinary systematic literature review [1,2]. A key finding was that most empirical studies had focused on short-term adoption of simple innovations by individual adopters. Studies of complex innovations (especially those requiring an organizational- or system-level adoption decision and a recurrent budget line); of the nonadoption and abandonment of innovations by individuals; and of local scale-up, distant spread, and long-term sustainability were sparse.

An update of that review in 2010 focused explicitly on organizational-level adoption and mainstreaming of technological innovations [3]. It identified some new literature on organizational-level routinization [4], but little new evidence on scale-up, spread, or sustainability—a finding that has been confirmed by other reviews since [5-9].

In recent years, technological innovation has moved apace and is now widely viewed as a significant potential contributor to health and wealth [10]. Yet the track record of technology programs, especially those that require major changes in organizations or the wider care system, is poor because of the combined problems of nonadoption and abandonment by individuals and difficulties with scale-up and spread [11]. While there is a growing general literature on the long-term sustainability of technology-supported change [12], studies of the sustainability of health and social care programs remain sparse.

These problems are illustrated by the paradox of telehealth (a term with contested definitions [13] but, broadly speaking, remote health care to the patient’s home). Despite much policy-level talk of triggering a revolution in service delivery and many small-scale proof-of-concept examples, telehealth services are rarely mainstreamed or sustained [14]. Nonadoption and abandonment of telehealth technologies by their intended users is common [15-17]. A nationwide audit in Norway showed that, despite geographical remoteness, a history of early adoption of telehealth, a strong policy push, and adoption in principle by 75% of all hospitals, fewer than 1% of outpatient consultations in participating specialties were actually undertaken via telehealth in 2013 [18].

Poor uptake of technological innovations is often explained in terms of barriers and facilitators. In a recent review of telehealth in heart failure, for example, we identified technology barriers, patient barriers, staff barriers, team barriers, business and financial barriers, and governance and regulatory barriers [13]. This list (and a reciprocal list of facilitators) resonates with other barriers-and-facilitators studies in the literature, including electronic patient record systems [7,19], electronic prescribing [20,21] and surgical safety checklists [22]. Such studies are a useful start, but they fall short of *theorizing* the failure to adopt, scale up, spread, or sustain a technology-supported program.

As our 2004 review of diffusion of innovations found, it is not individual factors that make or break a technology implementation effort but the *dynamic interaction between them*. The more complex an innovation or the setting in which it is introduced, the less likely it is to be successfully adopted, scaled up, spread, and sustained [7,23,24]. These interactions are unlikely to be elucidated by the randomized controlled trial design that still dominates much health technology research [25]. Rather, we need studies that are interdisciplinary, nondeterministic, locally situated, and designed to examine the recursive relationship between human action and the wider organizational and system context [25].

Among others, Lupton [26], May and Finch [27], Nicolini [28], Pols and Willems [29], Maniatopoulos et al [30], and our own team [31] have used different approaches to produce rich theorizations of the unfolding fortunes of technology-supported programs in health care. But such academic outputs are not directly accessible to the clinician on the ward, the manager in the office, or the executive in the boardroom—nor, indeed, to the patient in his or her home. Other authors (whose work is summarized in the Results section below) have drawn on such literature to produce unifying frameworks aimed at informing the work of implementation, although no previous framework has focused explicitly on nonadoption, abandonment, scale-up, spread, or sustainability.

We aimed to produce an evidence-based, theory-informed, but also accessible and usable framework that would enable those seeking to design, develop, implement, scale up, spread, and sustain technology-supported health or social care programs to identify and help address the key challenges in different domains and the interactions between them.

## Methods

### Study Design

**Figure 1:**
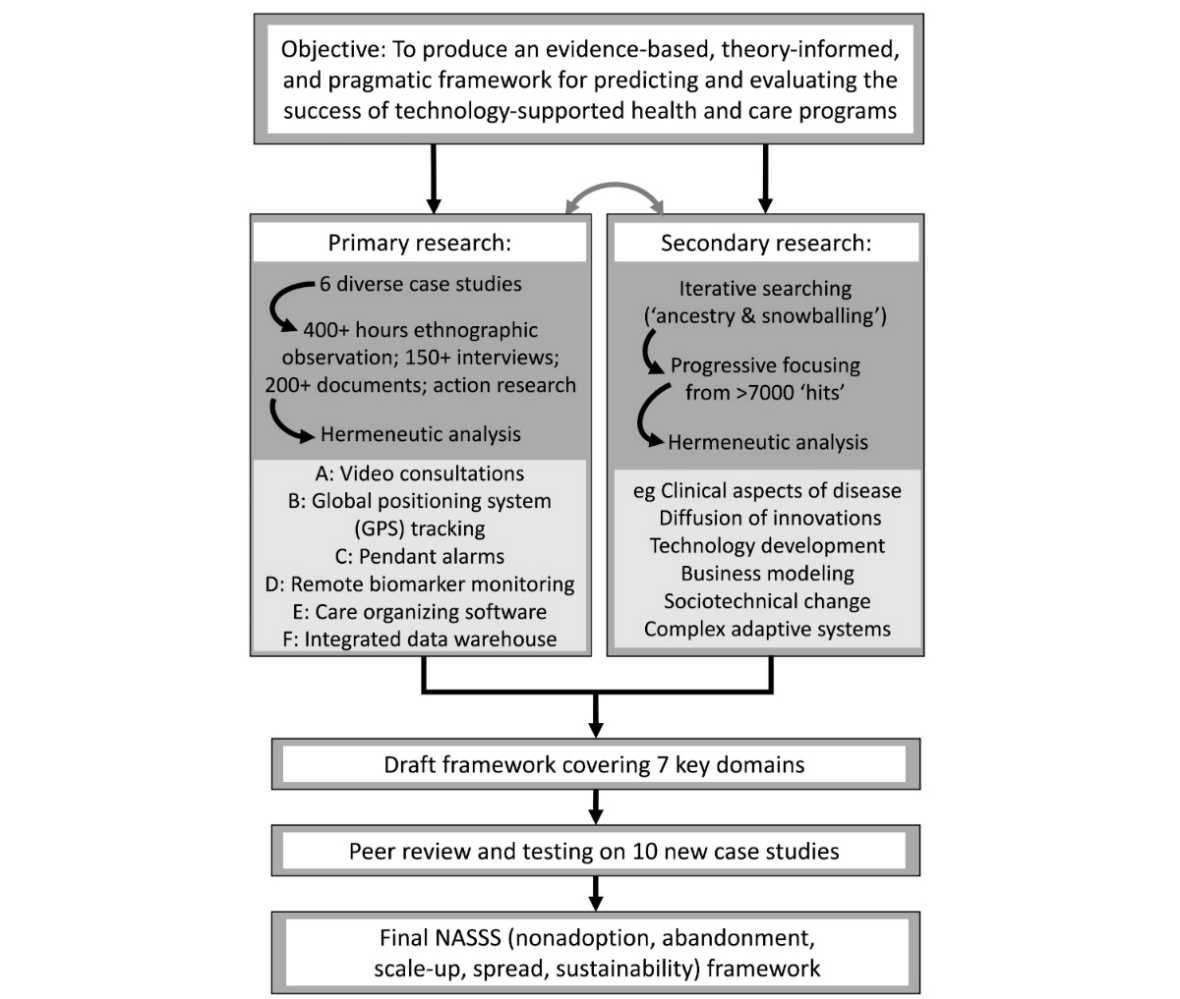
Study flowchart.

Figure 1 summarizes our design and methodology. The study had 2 parallel components: (1) secondary research (hermeneutic systematic review) to identify key domains and interactions, and (2) empirical case studies of technology implementation to explore, test, and refine these, followed by a synthesis phase, and peer review and refinement of the draft framework.

### Primary Research: 6 Empirical Case Studies

We selected a diverse sample of case studies from 2 research programs: Virtual Online Consultations: Advantages and Limitations (VOCAL) and Studies in Co-creating Assisted Living Solutions (SCALS), whose detailed methodology and ethical approval have been described elsewhere [25,32]. VOCAL (2015-2017, with an earlier set-up phase from 2011) was an in-depth study of the development, introduction, and local rollout of remote (video) consultations across 3 contrasting clinical specialties in a large, multisite UK hospital trust [32]. SCALS (2015-2020, with some data collected from 2013) is an action research study of the challenges faced by UK health and social care organizations who introduce technology-supported new service models; it includes examples from health care (eg, remote biomarker monitoring, video consultations, technologies for integrating care across organizations) and social care (safety alarms, global positioning system [GPS] tracking, care organizing apps) [25].

Case studies in VOCAL and SCALS involved qualitative interviews (with patients, clinicians, managers, technical designers, commercial partners, and—where relevant—investors), analysis of documents (correspondence, business plans, clinical records), ethnography (of technology use by patients or clients and staff, of meetings and events, and of technology design and functionality), and video recording of both ends of remote consultations [25,32]. Table 1 summarizes the subset of data from VOCAL and SCALS used for this study.

**Table 1:** Summary of data sources used in this analysis.

Study site or sites		Technology or technologies	Participants	Data sources
**Video outpatient consultations**				
	1A. Acute hospital trust (3 specialties—diabetes, prenatal diabetes, cancer—on different sites) 1B. Nurse-led heart failure service run from community hospital	Skype (acute hospital) and FaceTime (community hospital)	1A. 24 staff (9 clinicians, 10 support staff, 5 managers); 30 patients 1B. 10 staff (8 nurses, 1 manager, 1 administrator); 8 patients Plus 48 national stakeholders and wider informants on remote consulting	35 formal semistructured interviews plus ~100 informal interviews; ≥150 hours of ethnographic observation; 40 videotaped remote consultations (12 diabetes, 6 prenatal diabetes, 12 cancer, 10 heart failure); ≥500 emails; 30 local documents such as business plans, protocols; 50 national-level documents
**GPS^a^tracking for cognitive impairment**				
	2A. Social care organization in deprived borough in inner London, UK	GPS tracking devices supplied by 5 different technology companies, includes GPS tracking with virtual map and geofence alert functions	7 index cases; 8 lay caregivers; 5 formal caregivers; 3 social care staff; 3 health care staff; 3 call center staff	22 ethnographic visits and “go-along” interviews with index cases (~50 hours); 15 ethnographic visits with health and social care staff; 6 staff interviews; 5 team meetings; 3 local protocols
**Pendant alarms**				
	3A. Health care commissioning organization in deprived borough in outer London, UK 3B. Social care organization in mixed borough in the Midlands, UK	In both sites, pendant alarms and base units were supplied by multiple different technology companies and supported by local councils, each with a different set of arrangements with providers and an “arms-length management organization” alarm support service	Site 3A. 8 index cases; 7 lay caregivers; 12 professional staff Site 3B. 11 index cases; 9 health and social care staff from frontline service delivery to senior board level; 3 representatives from telecare industry	50 semistructured and narrative interviews; 61 ethnographic visits (~80 hours of observation) including needs assessments and reviews; 20 hours of observation at team meetings
**Remote biomarker monitoring in heart failure**				
	Acute hospital trusts in 6 different cities in United Kingdom	Tablet computer and commercially available sensing devices (blood pressure monitor, weighing scales, pulse oximeter)	7 research staff, including principal investigator and research coordinator for SUPPORT-HF^b^ trial; 7 clinical staff involved in trial; 4 clinical staff not involved in trial; (to date) 18 patient participants and 1 spouse	1 patient focus group; 8 patient interviews; 24 additional semistructured interviews; SUPPORT-HF study protocol and ethics paperwork; material properties and functionality of biomarker database
**Care organizing software**				
	5A. Health care commissioning organization in northern England 5B. National caregiver support charity in UK	5A. Web-based portal developed by small technology company for use by families to help them organize and coordinate the care of (typically) an older relative 5B. Smartphone app codesigned by caregiver support charity for same purpose	Product A: 2 technology developers and CEO^c^ of technology company; 4 social care commissioners; 30 health and social care staff considering using the device; 4 users of the device, 1 nonuser. Product B (to date): 2 members of care charity (including CEO); 10 qualitative case studies of users undertaken by another academic team	22 semistructured and narrative interviews; 16 hours’ ethnographic observations of meetings; autoethnographic testing of functionality and usability of devices; secondary analysis of third-party evaluation of Product B
**Data warehouse for integrated case management**				
	1 acute hospital trust, 1 community health trust, 3 local councils, 3 health care commissioning organizations	Integrated data warehouse incorporating predictive risk modeling (in theory interoperable with record systems in participating organizations)	14 staff; 20 patient participants	14 semistructured interviews; 50 ethnographic visits (~80 hours); 12 hours’ shadowing community staff; 4 hours’ observation of interdisciplinary meetings; 12 local protocols or documents

^a^GPS: global positioning system.

^b^SUPPORT-HF: Seamless User-Centred Proactive Provision of Risk-Stratified Treatment for Heart Failure.

^c^CEO: chief executive officer.

### Secondary Research: Hermeneutic Literature Review

Articles describing technology implementation frameworks and their applications were eligible if they (1) studied a technology that was perceived as new by intended users, (2) aimed (through the uptake and use of the technology) to improve service efficiency, or patient or client outcomes in health or social care; and (3) offered some kind of conceptual or theoretical framework. We were particularly interested in patient-facing technologies such as telehealth, but we also assessed other frameworks (eg, for health information systems) for transferable insights.

We began by selecting relevant studies from our hermeneutic literature review of telehealth in heart failure (covering 32 previous systematic reviews and 60 additional articles, including many that covered conditions beyond heart failure) [13]. We searched the reference lists of key studies [33-41]; we also put their titles into Google Scholar to identify 160 articles (surprisingly few) that had cited them subsequently, and manually screened these titles for relevance. We chose this “ancestry and snowballing” approach because initial database searching proved neither sensitive nor specific [42].

Having obtained few hits, we extended our search to the wider literature by tracking our original 2004 diffusion of innovations review [1]; we manually screened the titles and abstracts of over 4500 publications that had cited it. We did the same with 8 other highly cited reviews on the broader topic of innovation in health care [4-9,27,43] (around 3000 additional hits), using progressive focusing to limit the dataset. We favored authoritative reviews and added selected primary studies (characterized by strong theory, naturalistic methods, and rich detail, and including a focus on technology implementation). Where articles cited a specific theory, we obtained the original article describing that theory.

We used a simple data extraction form to summarize key aspects of each study (both theoretical and empirical). Using the hermeneutic (interpretive) methodology described in detail previously [13], we combined the findings of primary studies and previous reviews to generate a preliminary list of domains, potential interactions, and theoretical mechanisms.

### Synthesis and Framework Development

All 6 case studies generated large amounts of qualitative and quantitative data, not all of which was relevant to the objective of this study. Our first task was to delineate a more focused dataset of individual index cases (patients or clients) along with relevant staff interviews, field notes, and background documents (Table 1). For each case study, we analyzed qualitative data thematically and produced an initial narrative summary of the case, which we refined in the light of emerging theoretical evidence from the literature review. We conducted data analysis of the empirical case studies in parallel with progress on the hermeneutic literature review; each influenced the other. Findings broadly coalesced around key domains of influence (the patient, the technology, staff, and so on), which informed the development of an initial framework and raised further questions about the implementation process.

### Refinement, Peer Review, and Testing

We developed initial versions of the nonadoption, abandonment, scale-up, spread, and sustainability (NASSS) diagram and framework part way through the empirical work to guide our action research and inform cross-case theorization. As we applied the framework to real cases, challenges occurred that were not covered by it (eg, patients unable to use technologies because of comorbidities), so we searched more specifically for articles to inform additional domains and questions. We shared a near-final version of the framework with colleagues involved in 10 further large-scale technology-supported change programs (including email and video consultations in primary care; an online peer support network for people with mental health needs; remote biomarker monitoring in transplant patients; and an online tool for people to identify local services appropriate to their health and care needs); we further refined the framework in the light of their feedback.

## Results

Table 1 shows the datasets for our empirical case studies. Below, we give a brief overview of the cases before summarizing our literature review and introducing the NASSS framework.

### Empirical Case Studies

#### Case A: Video Outpatient Consultations

This case included 4 clinical services: 3 hospital-based services from VOCAL (young adult diabetes, prenatal diabetes, and cancer surgery, all using Skype; SS, unpublished data, 2017) and 1 community-based from SCALS (a nurse-led heart failure service run from 4 community hospitals, using predominantly FaceTime). In each, patients judged “appropriate” for video consultations by the doctor or nurse were offered this option. National policy makers viewed video consulting as a way of delivering health care efficiently to an aging population with rising rates of chronic illness. But the reality of establishing such services in busy and financially stretched public sector organizations proved far more complex and difficult than anticipated; progress was slow and required multiple organizational workarounds. Technical challenges in setting up video consultations with patients were typically mundane but potentially prohibitive (eg, forgotten passwords, poor connectivity, outdated software). When clinical, technical, and practical preconditions were met, video consultations appeared safe and were popular with both patients and staff, although only some clinicians agreed to participate.

By the end of the study period, video consultations had been abandoned in the prenatal diabetes service and put on hold in the community heart failure service, but the young adult diabetes and cancer surgery services were conducting around 20% of follow-up consultations remotely. In the (extremely busy) prenatal diabetes clinic, video consultations aligned poorly with a context involving multidisciplinary teams (patients were typically seeing multiple clinicians across departments) for a relatively short-term but high-risk condition and in the absence of integrated records (paper medical notes were held by the patient so not physically at hand for the clinician). In the heart failure clinic, the physical examination (eg, heart rhythm, leg edema) that the nurses considered essential was not easy in the remote environment (although sometimes possible with patient and caregiver assistance); multimorbidity and polypharmacy were common and in most cases the perceived risks and uncertainties associated with remote consulting were considered to outweigh the benefits.

#### Case B: GPS Tracking in Cognitive Impairment

Electronic tracking through GPS is used to monitor people with cognitive impairment who “wander” outside the home. We worked with a public sector social care organization to implement and adapt GPS tracking devices and a linked monitoring service for such individuals (of whom 11 were considered eligible and 7 assented). In what were typically very complex care contexts, GPS devices were useful to the extent that they aligned with a wider sociotechnical care network that included lay caregivers, call centers, and health and social care professionals. In this context, “safe” wandering was a collaborative accomplishment that depended on the technology’s material features, affordances, and aesthetic properties; a distributed knowledge of the individual and the places they wandered through; and a collective and dynamic interpretation of risk. Each index case required a high degree of tinkering (including customization of the device, liaison with the technology supplier, and adjustment of work routines) to achieve a solution that was acceptable. Despite this, only 3 individuals were still using the technology by the end of the 18-month study period.

#### Case C: Pendant Alarms

Pendant alarms (worn around the neck or on a wrist strap and connected to a remote call center) were the only patient-facing technology in widespread use in our dataset. Both study sites had a well-established sociotechnical infrastructure that included a named care team with expertise and local knowledge. Supply of a pendant alarm was typically initiated by a public sector organization and involved a local technology supplier to fit it, with or without support from an age charity. Clients could also self-refer. The setup usually depended on a network of lay caregivers available to respond to a summons; a safe box was usually installed containing a key so the rescuer could let themselves in, or an emergency response (eg, ambulance or 24/7 social care) was summoned as required. Users paid a set-up fee (around £25; US $40) plus a small weekly support fee (around £4.50; US $7), although some local care providers offered this service free of charge (eg, to people in receipt of welfare benefits). In almost all cases, the individual had multiple and complex needs (physical, cognitive, social) and was using multiple technologies in addition to the alarm [44].

In many but not all cases, activation of the pendant alarm led to help arriving promptly. On some occasions, there was a mismatch: the alarm was triggered when there was no objective need (perhaps by accident) or, more commonly, not triggered when caregivers felt it should have been—because the individual did not want to trouble anyone, did not believe the problem was serious or urgent or was unable to activate the device (eg, during a fit), or was not wearing the alarm at the time of the crisis. Sometimes, call center operators made judgments and put in “emotional work” to support the caller without alerting their relative or reassured them while help was on its way [45]. In 1 site, use of pendant alarms had evolved in that some lonely people (especially those with cognitive impairment) were being *encouraged* to press the alert button and talk to a call center operator even when there was no emergency, in order to reduce call-outs of the emergency services.

#### Case D: Remote Biomarker Monitoring (Telehealth)

This case study involved cardiology departments in 6 UK hospitals, each implementing biomarker monitoring (weight, blood pressure, heart rate) for heart failure as part of a multicenter randomized controlled trial (Seamless User-Centred Proactive Provision of Risk-Stratified Treatment for Heart Failure [SUPPORT-HF]). The tablet technology supplied to patient participants had been developed using a codesign methodology [46]. Participants in both arms of the trial received the technology and automated feedback messages (eg, if results went outside preset parameters); in the intervention arm, the patient’s family physician was alerted to out-of-range results and offered suggestions for changes in therapy, whereas in the control arm, results were made available on a Web portal for the patient’s physician to access if they chose to. Staff at the different SUPPORT-HF sites engaged variably with the study, sometimes leading to slower than predicted recruitment. A minority of clinicians were reluctant to refer patients or engage with the trial protocol, citing “previous bad experiences with telehealth,” concern that a remote monitoring service would threaten their jobs, or a perception that patients “deserved better.” Patient participants expressed a range of views about remote biomarker monitoring; some took an active interest in their readings, engaged enthusiastically with the feedback they received, and found this monitoring reassuring. Others found the experience confusing and did not know (or wish to know) what the numbers meant. In some cases, a research nurse known to the participants provided (unofficial) telephone support to maintain engagement. Another problem with the remote monitoring service was the variability of broadband speed outside the main cities, which meant that more than half of potentially eligible participants in 1 site could not be included in the study.

#### Case E: Care Organizing Software

This case study followed the very different fortunes of 2 software products, each designed to help relatives and friends (and sometimes professional staff as well) organize tasks and visits for someone with health or care needs. Product A, a Web portal, had been developed in-house by a small software company, based on a previous caring experience by one of the company staff. The business model was to sell the product to care organizations who would then provide it to their clients for free. The developer did not initially anticipate that either intended end users or participating care organizations would need any training or ongoing support to use the portal. Product A was not successful during the study period; fewer than 5 families were ever identified as actively using it.

Product B was a smartphone app (with linked Web portal) that had been developed via publicly funded research and development using codesign methodology by a national caregivers’ charity. The charity had previously identified a need for such software; they worked with a specialist app developer company and carefully selected pilot families. From the outset, the charity recognized that caregivers would need to be made aware of the product through mass mailing and to be actively invited and supported to use it, and that a helpdesk service would be needed. The app was made available commercially (via the App Store) for £2.99 (about US $5). Users signed up gradually but steadily; there was no tipping point, but at the time of writing over 1000 families are using the product through the care charity; in a preliminary evaluation, most spoke highly of it (and of the charity support).

#### Case F: Integrated Case Management Via Data Sharing

Case management is a way of organizing health and social care services through assessment and care planning by multidisciplinary teams with the aim of managing the growing challenge of emergency hospital admissions (and readmissions) in older people with multiple health and care needs. To avoid the high human and financial cost of such admissions, coordinated action and frequent dialogue between primary care providers, secondary care providers, and social care and other formal and informal caregivers is often needed. In the SCALS study, 1 site had introduced an integrated data warehouse incorporating a predictive risk modeling tool to automate the identification of people at high risk of hospital admissions through risk stratification, and to facilitate shared access to care plans in efforts to achieve integrated care. Although the data warehouse was part of business as usual in this site, in practice people at high risk of hospital admission were identified through a combination of risk stratification and clinical judgment. Care plans were shared in a range of ways, sometimes through the integrated data warehouse and sometimes bypassing it.

### Literature Review

Our search for evidence-based approaches to guide our empirical work on the above case studies identified 28 technology implementation frameworks, informed by several theoretical perspectives, which we sorted into a simple taxonomy [13,43,47-72]; see Multimedia Appendix 1.

A key limitation of many previous frameworks was the lack of detailed analysis of the condition or problem for which a new technology was [part of] the intended solution. Some assumed a “textbook” condition—simple, isolated, easily characterized, and amenable to management by algorithm or protocol using a one-size-fits-all (or minimally customizable) technology. Yet there is much empirical evidence that the health and care needs of real people are extremely heterogeneous, even when they have the “same” condition. For example, Tait et al’s case-by-case analysis of patients in a heart failure clinic found that every one of them required significant customization and ongoing adaptation of the care package recommended in the guideline [73].

A prominent policy prediction, typically couched in the language of “empowerment,” is that remote technology will make care more efficient by encouraging self-management of chronic conditions [74]. But as May et al have pointed out in their burden of treatment theory, shifting the work of care from clinic to community places new demands on the sick (and hence raises ethical questions) [75]. Depending on the condition, such work may also be physically or cognitively impossible. The anchored, realistic, cocreative, human, integrated, and evaluated (ARCHIE) framework derived from our earlier empirical work on assisted living technologies emphasized the diverse manifestations of multimorbidity and social care need; it recommends commencing with a realistic assessment of the nature (and likely progression) of the condition and a focus on what matters to the user [47].

No previous framework explicitly considered inequalities in access, uptake, and use of health and care technologies by age, sex, socioeconomic status, or ethnic group, although previous empirical studies have highlighted substantial differences across such groups [76]. Chronic health conditions and care needs are strongly patterned by social determinants. For example, type 2 diabetes, heart failure, depression, cognitive impairment, and general frailty are all more than twice as common in the poorest and least well-educated quintiles of society as in the richest and best educated [77]. The poor may also have less rich social networks, less reliable access to broadband, lower digital literacy, and greater likelihood of having problems such as debt or unsuitable housing [77,78].

Most previous frameworks addressed the material properties of the technology, such as its physical features, functionality, and interoperability. Few considered its symbolic properties (some technologies—such as mobile phones—have connotations of youth, progress, and friendship; others—such as GPS tracking devices or pendant alarms—symbolize dependence or external control). One or two frameworks considered what knowledge or skills (and hence training and support) were needed for intended users to be confident in operating the technology.

A question addressed tangentially or not at all by previous frameworks was *what kind of knowledge* does the technology generate? For example, telehealth technologies fall into two broad categories. Remote monitoring devices transmit objective biomarkers such as weight, blood pressure, and oxygen saturation, and responses to closed questions on symptoms and compliance, and perhaps also transmit instructions or educational messages (what Pols has called “cold” telehealth [79]). Remote communication devices create the possibility for more conventional conversations between patients and clinicians by telephone or video (“warm” telehealth). These different technologies bring very different kinds of knowledge into play and, by design or default, exclude other knowledge and influences from the frame.

Very few frameworks in our sample included an assessment of whether a technology was likely to be *worth* introducing—that is, its value proposition. “Value” means different things to different stakeholders; it has parallels to Rogers’ term “relative advantage’ (the extent to which a potential adopter believes that the innovation is better than what has gone before [80]). From the patient’s perspective, there is often a trade-off between the potential benefits of technologies, their costs (and the person’s willingness and ability to contribute to these), the work required to use them (and the person’s capacity to do so), and the desirability of medicalization and surveillance [81].

Lehoux et al distinguish between a health technology’s upstream value as viewed by investors (especially the business case for generating profits, further spin-offs, and highly qualified jobs), drug and device regulators (preliminary evidence of efficacy and safety), and financial regulators (auditable business processes and governance), and its downstream value as viewed by clinicians and policy makers (including its impact on patients and health care costs) [82,83]. Health technology development is often characterized by poor alignment between supply-side and demand-side value [82,84,85].

In previous frameworks, technology adoption by health care staff was most commonly theorized using Davis’s technology acceptance model, comprising perceived usefulness, perceived ease of use, and attitude toward the technology [86]; or Bandura’s social learning theory, the relevant aspect of which is that people learn by observing and imitating the behavior of others [87,88]. Critics of the technology acceptance model have argued that it fails to account for human and social change processes [89]. Sociological theories of technology adoption, which emphasize the norms and expectations associated with different social positions and professional groups [26,27,31], were not extensively used in previous frameworks, with the exception of May and Finch’s normalization process theory [27], of which relational integration—how the technology affects human relations such as the doctor-patient relationship—is one component.

In considering our own case studies, we were drawn to sociological theories because the new technologies often had implications for staff identity, professional commitments, and scope of practice. Acceptance by professional staff may be the single most important determinant of whether a new technology-supported service succeeds or fails at a local level [17,36,39,79,90,91]. Local champions appear key to persuading their peers that the technology-supported service is effective, safe, and “normal” (ie, professionally appropriate) [17,92]. We have previously developed a theoretical model of clinician resistance to new health care technology made up of 4 elements: resistance to the *policy* reflected in the technology (eg, a policy of shifting the work of disease management from professional to patient); resistance to the *sociomaterial constraints* (eg, clunkiness, dependability) of the new technology; resistance to *compromised professional practice* (eg, less scope for exercising judgment); and resistance to *compromised professional relationships* (eg, a perception that a remote interaction is less professional than a face-to-face one) [93].

One framework in our sample addressed technology acceptance by patients. The digital health engagement model was based on both burden of treatment theory and normalization process theory [48]. It proposes 4 key influences on whether an individual will engage with a health technology: personal agency and motivation (which is affected by aspects of their illness); personal life and values; the engagement and recruitment approach taken by those seeking to promote the technology; and the quality of the health technology.

Two frameworks drew on DeLone and McLean’s classic theoretical model of information system success [94]. This considers system quality, information quality, usage attributes, user satisfaction, individual impact, and organizational impact [49,50]. While this framework had some resonance with our empirical data, it did not address the patient-facing aspects of health and care technologies, nor did it address contextual influences or change over time (we classified it as a static framework), and hence did not help us with our study of scale-up, spread, and sustainability.

Surprisingly few frameworks considered the organizational setting. Some antecedent characteristics of organizations have been shown to support innovation at an organizational level [1]. These include a devolved organizational structure (with each department or unit able to make semiautonomous decisions), significant organizational slack (that is, spare resources that can be channeled into new projects), and strong leadership, good managerial relations, a risk-taking climate (staff are rewarded rather than punished for trying things out), opportunities for sense making (that is, collectively arguing out the meaning of an innovation [95]), and what is known as absorptive capacity: “a set of organizational routines and processes by which [organizations] acquire, assimilate, transform, and exploit knowledge to create a dynamic organizational capacity” [96]. These organizational determinants of innovation align with complexity theory’s emphasis on local adaptation and the need for creativity to address unique emerging issues [97]. A specific innovation is more likely to be taken up if there is strong tension for change, good innovation-system fit (that is, the innovation fits well with existing work and routines), widespread support for (and limited opposition to) the innovation, and systematic assessment of the implications [1].

Another important aspect of implementation omitted by most previous frameworks was the health or care organization’s business model for introducing the technology. This includes the resources to support the model, key partners and relationships, the transaction mechanism (how will the organization interact with the supplier?), the value structure (how and when will value, including benefits for patients, be created and investment costs recouped?), and organizational design issues (what changes in organizational structure and processes are required or presumed by the new technology?) [11,14]. A review by van Limburg et al highlighted the financial and business challenges associated with eHealth technologies, including their (typically) fragmented deployment, multiple stakeholders and interdependencies, lack of recognition of the ongoing work of implementation, and an overreliance on the results of experimental efficacy trials [11].

Most, but not all, previous frameworks considered how a new technology would fit with existing organizational routines (defined as recurrent, collective patterns of interaction that both coordinate and control organizational work [4]). Technologies create opportunities for developing new routines and care pathways, but they also disrupt existing patterns of team interaction in ways that can prove more complex than initially anticipated [98]. There is, almost inevitably, a crucial gap between the nuanced, flexible, and often unpredictable nature of human activity and what it is possible to deliver technically. This is especially crucial when considering something as complex and exception filled as clinical work (Case A) [99]. As Grudin (cited by Symon et al [100]) put it:

Work processes can be described in two ways: the way things are supposed to work and the way they do work. Software that is designed to support standard procedures can be too brittle.

page 25

Cherns’ classic theory of sociotechnical design, originally developed in the 1970s, is built on the principle that introducing technologies in an organization is a social process that depends on values, mindsets, and engagement, as well as on clear and extensive communication about what changes are occurring and why [101]. It is also an evolutionary process (sociotechnical systems are grown, not built), hence best achieved by early and active input of frontline workers into the [re]design of work routines—a principle that has long been recognized (but rarely adequately applied) in health care [100,102]. Also highly relevant to the health and care environment is what Weick called “technology as equivoque” [103] and Orlikowski (drawing on earlier work on the social construction of technology) called “interpretive flexibility” [104]: a technology introduced into an organization is open to multiple interpretations; successful embedding will require opening up a space for dialogue, listening to concerns, and allowing people time to argue out the challenges and learn from the experiences of others before a “closure” over possible interpretations is reached [88,105]. As Stewart and Williams [105] stated,

Innovation is not restricted to the prior design of an artefact, but continues as artefacts are implemented and used (innofusion). Supplier offerings are inevitably incomplete in relation to the complex, heterogeneous and evolving requirements of users; work needs to be done by specific users to incorporate these generic solutions to their particular contexts and practices (domestication).

page 195

Surprisingly, few previous frameworks in the health and social care literature have attempted to capture this insight, which partly explains why on-the-job training in technology use and ongoing helpdesk support are key to the implementation process [1].

A prominent theme in our empirical findings—but addressed tangentially or not at all by most previous frameworks—was that implementing health technology programs involves a great deal of work [106]. Normalization process theory unpacks implementation work into 4 categories: *coherence* (the work that people do to make sense of a practice), *cognitive participation* (work to enroll and engage other people in relation to that practice), *collective action* (work to enact the new practice, including efforts to bridge the model-practice gap described above), and *reflexive monitoring* (the work involved in assessing and adjusting a practice in use, including evaluating the impact of the technology and demonstrating its value to others) [27]. Implementation work may be particularly onerous in relation to health technologies because of the complexities and institutional challenges of addressing, for example, data security and patient privacy, interoperability across multiple information systems, resistance from health care professionals with a high degree of autonomy, and disruption to the critical granularity of clinical workflow [106]. First-order problems (such as slow technical performance) typically generate second-order problems, such as dramatically increased workloads (sometimes necessitating safety-critical workarounds), and perhaps third-order ones, such as reputational damage [106]. Failures in all 4 of May and Finch’s work categories were evident in a systematic review of reasons for unsuccessful telemedicine implementation informed by normalization process theory [107].

Around half of the frameworks identified in this review included a question on the wider context for technology-supported care [108]. New technologies generate technical—and commercial and political—questions around interoperability standards, customer lock-in, customizability, substitutability, supplier relations, and the marketplace; policy questions about mainstreaming and funding new models of care; professional and managerial questions around standardization of algorithms and protocols; financial questions about who pays for which aspect of a networked service; legal questions around intellectual property; regulatory questions about safety, efficacy, and good business processes; and jurisdictional questions around liability, licensing, and the management of health information in shared environments [108-113]. The industry impetus of agile, rapid-iteration technology development and the “fail early, fail often” principle typically followed for software products contrasts with the risk-averse, highly regulated, and randomized trial-dominated context of much biomedical innovation [11,82,114].

While some implementation frameworks were designed around a rigid (and apparently systematic) logic model [51], we have previously argued that such an approach is counterproductive because eHealth technologies are typically introduced into a complex system in a turbulent and contested policy context [52]. Complexity principles distinguish simple phenomena (straightforward, predictable, few components) from complicated (multiple interacting components or issues) or complex (dynamic, unpredictable, not easily disaggregated into constituent components) ones [97]. Chambers et al’s dynamic sustainability framework recognizes that, in order to be sustained, an innovation must adapt to its unique local environment and evolve over time [115], echoing Hawe et al’s conceptualization of interventions as “events in [complex] systems” [116]. Several other recently published technology implementation frameworks have embraced complexity theory and argued strongly for a developmental, contextualized, and adaptive approach [43,47,53-60].

Abbott et al [60], for example, recommend that because the “same” program will play out differently in different contexts (and in the same context over time), collecting data at multiple levels, from multiple sites and longitudinally, will help elucidate these contextual influences. Because adaptation is key to embedding, inflexible milestones and overzealous measures of fidelity should be avoided. Local champions are likely to be key to participatory and developmental approaches; they should be identified early and partnered over time. Also key to the achievement of sustainability is attention to penetration (the extent to which the technology and its use become integrated with workflows so that workarounds are no longer necessary).

Van Gemert-Pijnen et al reviewed 16 frameworks for the implementation of eHealth innovations published up to 2009 (although in our own classification, 3 of these were not actually frameworks) [43]. They produced a multilevel theorization including diffusion of innovations, technology acceptance, service improvement, and organizational development. Their holistic technology implementation framework, known as the Centre for eHealth & Wellbeing Research roadmap, comprises 5 overlapping stages undertaken by a multidisciplinary team with iterative formative evaluation of each: (1) contextual inquiry (information gathering about the users and the environment, including ethnography and the use of scenarios); (2) value specification (including economic, social, and behavioral dimensions); (3) design (building prototypes that fit with values and user requirements); (4) operationalization (introduction and employment of the technology, including implementing a business model); and (5) summative evaluation (of uptake and impact).

Van Dyk, writing from an industrial engineering background, produced a taxonomy of eHealth implementation frameworks based on several theories, including diffusion of innovations, technology acceptance and use, e-readiness of organizations, transactional economics, and the information system life cycle, as well as considering more pragmatic barriers-and-facilitators studies [59]. She proposed a holistic approach that includes “technology, organizational structures, change management, economic feasibility, societal impacts, perceptions, user-friendliness, evaluation and evidence, legislation, policy and governance.”

Van Dyk drew an important insight from organizational life cycle studies (including her own work on a telemedicine service maturity model for health care organizations [58])—that the challenges and constraints of technology-supported services vary with stage of development [59]. That is, (1) at the prototype stage, the main emphasis is on proof of concept and usability; (2) in small-scale pilots, it is on staff and societal acceptance (typically with an emphasis on the evidence base); (3) at the stage of wider local rollout—when financial support typically moves from external research grant to real set-up costs and a recurrent budget line—it is on financial and organizational considerations; and (4) when being considered for national rollout, it is on regulation, standardization, and security.

One final concept, which appears key to organizational adaptation over time but was not explicitly addressed in any of the previous frameworks, is the notion of resilience, defined as “the intrinsic ability of a system to adjust its functioning prior to, during, or following changes and disturbances so that it can sustain required operations, even after a major mishap or in the presence of continued stress” [117]. Organizational psychologists emphasize the importance of macrocognition: that is, reflecting collectively and continuously about how the organization is responding to change, including ongoing sense making, detecting critical events, and coordinating adaptive actions [118,119]. Introduction of new health care information technology systems can lead to loss of system resilience, since new technologies intended to automate work and assure safety may have the unintended effect of reducing time for collaborative dialogue, masking key trends in data (perhaps through information overload and loss of overview), making work routines brittle, and bypassing clinical judgment [118,120]. Cho et al have warned that the study of resilience in relation to new technologies requires multilevel analysis and is fraught with paradoxes (eg, that developing resilience in one part of the system may generate brittleness in another) [121].

In sum, we found the integrated frameworks of van Gemert-Pijnen et al [43] and Van Dyk [59] extremely helpful and used them as the starting point for analyzing our own dataset, modifying and refining them in the light of other high-quality frameworks published subsequently [53-55,60], and adding additional theoretical concepts (eg, burden of disease, health literacy, organizational resilience) and insights from our empirical data (especially our findings on the diverse and idiosyncratic nature of many conditions, which had received little attention in any previous framework).

### Synthesis: The NASSS Framework

**Figure 2:**
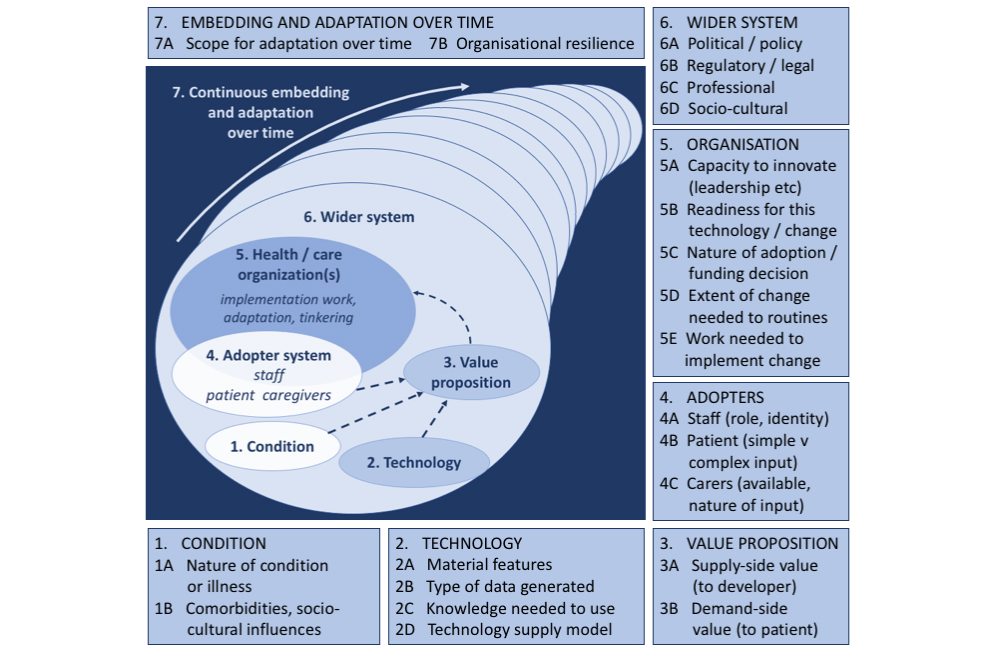
The NASSS framework for considering influences on the adoption, nonadoption, abandonment, spread, scale-up, and sustainability of patient-facing health and care technologies.

The final version of the NASSS technology implementation framework is shown in Figure 2 and expanded in Table 2. It consists of 13 questions in 6 domains: the condition, the technology, the value proposition, the adopter system (staff, patient, and lay caregiver[s]), the health or care organization(s) (including attention to the work of implementation and adaptation), and the wider (institutional and societal) context. It also includes a seventh domain that considers interactions and adaptations over time. The framework is intended to be used reflexively to guide conversations and help generate ideas, not as a checklist.

Our case studies raised a variety of challenges across all 7 domains, each of which could be classified as simple (straightforward, predictable, few components), complicated (multiple interacting components or issues), or complex (dynamic, unpredictable, not easily disaggregated into constituent components) [97]. Programs characterized by complicatedness proved difficult but not impossible to implement. Those characterized by complexity in multiple NASSS domains rarely, if ever, became mainstreamed. Multimedia Appendix 2 gives examples from our case studies in each of the domains, which we consider in turn.

#### The Condition

This domain addresses the clinical (question 1A) and the comorbidities and sociocultural aspects (question 1B) of the condition. It reflects the striking finding across all our case studies that only a fraction of potential end users were assessed by their clinicians as “suitable” for the technology. In the majority, the condition was considered clinically high risk, unpredictable, or atypical (eg, complicated by comorbidities or sociocultural factors, especially cognitive or health literacy considerations).

#### The Technology or Technologies

Question 2A addresses material and technical features of the technology. The technologies in our case studies were more or less freestanding. In all cases, features such as size, sounds, aesthetics, and “clunkiness” had significant impact on the technology’s actual and perceived usability and appropriateness. Many were insufficiently prototyped. Dependability of technologies was key, especially in high-risk conditions or social situations.

Question 2B considers the knowledge generated or made visible by technology. This includes not only the accuracy of the data but also the extent to which those data are accepted, trusted, and considered sufficient for decision making. Engaging with the data generated by patient-facing technologies may inform, educate, and empower patients and lay caregivers. But data may also be misinterpreted by the patient or cause distress.

Question 2C addresses the knowledge and support needed to use the technology. Some technologies are much easier to operate than others; some require frequent troubleshooting; and some assume a different organizational role—or even an altered professional identity—for the user. Some patient-facing technologies require no knowledge form the patient; others require clinical knowledge, technical knowledge, and the ability to make judgments about (for example) what counts as urgent.

Question 2D addresses issues for sustainability raised by the technology supply model—that is, how the technology was procured, the nature of the client-supplier relationship, and the level of potential substitutability via the marketplace. The telehealth device marketplace is notorious for its lack of interoperability as companies attempt to retain their market share through lock-in of customers. The consequences for health and social care services—and their clients—of market withdrawal could be significant. While procurement options may not have immediate impact on scaling up and sustainability, they are likely to have significant impact in the long term and may influence decisions on whether to adopt an innovation in the first place. Customizable, off-the-shelf technologies (COTS) [113] offer relatively low cost (as they benefit from economies of scale) but may not be customizable to the extent that users really need (see domain 1). In contrast, bespoke solutions offer better fit with users’ needs but at higher cost (both developmental and ongoing). Both options may be subject to risks of supplier withdrawal from the marketplace, with small and medium-sized enterprises being especially vulnerable.

#### The Value Proposition

This domain concerns whether a new technology is worth developing in the first place—and for whom it generates value. Question 3A addresses upstream value, which follows the supply-side logic of financial markets and investment decisions (and hence depends on preliminary tests of efficacy and safety, and evidence of good business practice). Question 3B addresses downstream value, which follows the demand-side logic of health technology appraisal, reimbursement, and procurement (ie, relates to evidence of benefit to patients and real-world affordability). As Multimedia Appendix 2 illustrates, our dataset included telling examples of mismatch between supply-side and demand-side value.

**Table 2:** Domains and questions in the nonadoption, abandonment, scale-up, spread, and sustainability (NASSS) framework.

Domain/question		Simple	Complicated	Complex
**Domain 1: The condition or illness**				
	1A. What is the nature of the condition or illness?	Well-characterized, well-understood, predictable	Not fully characterized, understood, or predictable	Poorly characterized, poorly understood, unpredictable, or high risk
	1B. What are the relevant sociocultural factors and comorbidities?	Unlikely to affect care significantly	Must be factored into care plan and service model	Pose significant challenges to care planning and service provision
**Domain 2: The technology**				
	2A. What are the key features of the technology?	Off-the-shelf or already installed, freestanding, dependable	Not yet developed or fully interoperable; not 100% dependable	Requires close embedding in complex technical systems; significant dependability issues
	2B. What kind of knowledge does the technology bring into play?	Directly and transparently measures [changes in] the condition	Partially and indirectly measures [changes in] the condition	Link between data generated and [changes in] the condition is currently unpredictable or contested
	2C. What knowledge and/or support is required to use the technology?	None or a simple set of instructions	Detailed instruction and training needed, perhaps with ongoing helpdesk support	Effective use of technology requires advanced training and/or support to adjust to new identity or organizational role
	2D. What is the technology supply model?	Generic, “plug and play,” or COTS^a^ solutions requiring minimal customization; easily substitutable if supplier withdraws	COTS solutions requiring significant customization or bespoke solutions; substitution difficult if supplier withdraws	Solutions requiring significant organizational reconfiguration or medium- to large scale-bespoke solutions; highly vulnerable to supplier withdrawal
**Domain 3: The value proposition**				
	3A. What is the developer’s business case for the technology (supply-side value)?	Clear business case with strong chance of return on investment	Business case underdeveloped; potential risk to investors	Business case implausible; significant risk to investors
	3B. What is its desirability, efficacy, safety, and cost effectiveness (demand-side value)?	Technology is desirable for patients, effective, safe, and cost effective	Technology’s desirability, efficacy, safety, or cost effectiveness is unknown or contested	Significant possibility that technology is undesirable, unsafe, ineffective, or unaffordable
**Domain 4: The adopter system**				
	4A. What changes in staff roles, practices, and identities are implied?	None	Existing staff must learn new skills and/or new staff be appointed	Threat to professional identity, values, or scope of practice; risk of job loss
	4B. What is expected of the patient (and/or immediate caregiver)—and is this achievable by, and acceptable to, them?	Nothing	Routine tasks, eg, log on, enter data, converse	Complex tasks, eg, initiate changes in therapy, make judgments, organize
	4C. What is assumed about the extended network of lay caregivers?	None	Assumes a caregiver will be available when needed	Assumes a network of caregivers with ability to coordinate their input
**Domain 5: The organization**				
	5A. What is the organization’s capacity to innovate?	Well-led organization with slack resources and good managerial relations; risk taking encouraged	Limited slack resources; suboptimal leadership and managerial relations; risk taking not encouraged	Severe resource pressures (eg, frozen posts); weak leadership and managerial relations; risk taking may be punished
	5B. How ready is the organization for this technology-supported change?	High tension for change, good innovation-system fit, widespread support	Little tension for change; moderate innovation-system fit; some powerful opponents	No tension for change; poor innovation-system fit; many opponents, some with wrecking power
	5C. How easy will the adoption and funding decision be?	Single organization with sufficient resources; anticipated cost savings; no new infrastructure or recurrent costs required	Multiple organizations with partnership relationship; cost-benefit balance favorable or neutral; new infrastructure (eg, staff roles, training, kit) can mostly be found from repurposing	Multiple organizations with no formal links and/or conflicting agendas; funding depends on cost savings across system; costs and benefits unclear; new infrastructure conflicts with existing; significant budget implications
	5D. What changes will be needed in team interactions and routines?	No new team routines or care pathways needed	New team routines or care pathways that align readily with established ones	New team routines or care pathways that conflict with established ones
	5E. What work is involved in implementation and who will do it?	Established shared vision; few simple tasks, uncontested and easily monitored	Some work needed to build shared vision, engage staff, enact new practices, and monitor impact	Significant work needed to build shared vision, engage staff, enact new practices, and monitor impact
**Domain 6: The wider context**				
	6A. What is the political, economic, regulatory, professional (eg, medicolegal), and sociocultural context for program rollout?	Financial and regulatory requirements already in place nationally; professional bodies and civil society supportive	Financial and regulatory requirements being negotiated nationally; professional and lay stakeholders not yet committed	Financial and regulatory requirements raise tricky legal or other challenges; professional bodies and lay stakeholders unsupportive or opposed
**Domain 7: Embedding and adaptation over time**				
	7A. How much scope is there for adapting and coevolving the technology and the service over time?	Strong scope for adapting and embedding the technology as local need or context changes	Potential for adapting and coevolving the technology and service is limited or uncertain	Significant barriers to further adaptation and/or coevolution of the technology or service
	7B. How resilient is the organization to handling critical events and adapting to unforeseen eventualities?	Sense making, collective reflection, and adaptive action are ongoing and encouraged	Sense making, collective reflection, and adaptive action are difficult and viewed as low priority	Sense making, collective reflection, and adaptive action are discouraged in a rigid, inflexible implementation model

^a^COTS: customizable, off-the-shelf.

#### The Adopter System (Staff, Patient, Caregivers)

Question 4A is about adoption (and continued use) of the technology by staff. Reflecting the findings of previous studies reviewed above, some staff in each of our case studies simply did not engage with the program or use the technology. Nonadoption (or in some cases, abandonment) of the technology was occasionally explained by the technology’s attributes (such as usability or ease of use). More commonly, staff were concerned about threats to their scope of practice or to the safety and welfare of the patient—and even, in some cases, about a fear of job loss.

Question 4B addresses adoption by patients or clients, including acceptance (hence symbolic meaning and aesthetics) and the work required of them.

Question 4C addresses the assumptions that may be built into the technology (or the linked service model) about the availability and behavior of lay caregivers. We encountered many cases of nonuse of all patient-facing technologies that were explained by weak or absent social networks, limited information technology skills (and distrust of technology) among lay caregivers, or long-standing family conflicts, which the technology sometimes brought to the surface but never solved (see examples in Multimedia Appendix 2).

#### The Organization(s)

Questions 5A and 5B address the organization’s capacity (to embrace any service-level innovation) and readiness (for a specific technology), respectively. Our case studies included a wide range of antecedent conditions and levels of readiness that influenced uptake and internal scale-up of technology-supported programs (see examples in Multimedia Appendix 2).

Question 5C addresses the adoption decision, typically a board-level decision to allocate a budget line to support a particular technology. Our empirical data indicated three main problems with business modeling. The first problem was lack of data: it was often impossible to predict the uptake, use, and impact of the technology or the amount of investment needed to get (and keep) it up and running. Predictions were often guesstimates and typically overoptimistic, for example, in terms of numbers of potential users and potential costs and efficiency savings. The second problem was lack of money to support the program (see “organizational slack” above). Indeed, several organizations in our case studies did not appear to even recognize the need for a dedicated budget, over and above the cost of the technology, to support implementation and maintenance. The third problem was interdependencies between organizations and the teams working within and across them.

Question 5D considers the extent to which established work routines will be disrupted or made too brittle by the new technology. In some cases, there will be a transition period in which new collaborative routines linked to the new technology will be played out in parallel with existing routines—perhaps on legacy systems—before (hopefully) replacing them.

The last question in domain 5 concerns the *work* involved in implementation. All 6 case studies affirmed previous research that such work is extensive, often hidden and typically underestimated at the planning stage [122]. Our ethnographic data affirmed what Pols and Willems showed previously [29]: that technologies must be “tamed” in any particular setting by careful attention to the fine-grained detail of context (a process Pols and Willems called “tinkering” and Stewart and Williams called “domesticating” [105]). There is also the need for work on what Weick called sense making [95,103] and May and Finch called “coherence work” [27]: to make collective sense of the technology in the organizational setting and build a shared vision of its potential (including a realistic assessment of what the technology *cannot* do).

#### The Wider Context

Question 6A relates to the wider institutional and sociocultural context, which in our case studies was often key to explaining an organization’s failure to move from a successful demonstration project (heavily dependent on particular champions and informal workarounds) to a fully mainstreamed service (scale-up) that was widely transferable (spread) and that persisted long term (sustainability). Aspects of wider context that proved pivotal in our case studies included health policy (including which service models were formally approved for funding—see example in Multimedia Appendix 2 of the difficulties in securing a nationally approved tariff for remote consultations), fiscal policy (the overall amount of funding available locally and nationally for health and care provision), the position taken by professional bodies and defense societies (who de facto defined what was acceptable professional practice), and legal and regulatory aspects of patient-facing technology development.

#### Interaction Between Domains and Adaptation Over Time

While the domains above can be distinguished analytically, the reality of any technology implementation project is that at an empirical level they are inextricably interlinked and dynamically evolving, often against a rapidly shifting policy context or continued evolution of the technology (and, at an individual patient level, as the condition deteriorates or fluctuates over time).

Question 7A relates to the medium- and long-term feasibility of continuing to adapt the technology and the program. Some technologies were more amenable to adaptation than others. Adaptation of staff roles and care pathways was also difficult when organizations were in subcontractor relationships (sometimes with private providers).

Question 7B concerns organizational resilience, and particularly what May and Finch called “reflexive monitoring” [27] and Patterson et al called the “macrocognitive functions” of sense making, including in particular the ability to detect critical events or issues and respond to these through coordinated action [118].

While an adaptive and reflexive approach is essential for effective scale-up, spread, and sustainability, it is impossible to be prescriptive at the outset as to how to go about this. Both formative and summative evaluation of the program’s success will need to use imagination and multiple methods, along with a narrative account of what happened and why, in order to capture its multiple interdependencies, nonlinear effects, and unintended consequences [123].

### Results of Prospectively Testing the NASSS Framework

Colleagues found our near-final NASSS framework helpful in considering challenges to implementation of a range of technology-supported health or care programs. While some used the framework (as we ourselves did) prospectively and in real time to predict and explore the challenges of implementing an existing technology to support a new program, some used it retrospectively to explain failures and partial successes in past projects. One group (a design company) suggested that the NASSS framework might be used at an early (design) stage to focus attention first on the condition and adopter system domains (ie, the needs of intended users), which would then inform both the technology and value proposition domains.

## Discussion

### Principal Results

Building on previous work by other groups, this study has developed and applied a new framework for predicting and evaluating the success of technology-supported health and social care programs. Across a diverse sample of 6 empirical case studies followed (so far) for up to 3 years and tested briefly (for what might be called face validity) in a further sample of 10 additional case studies, we identified and explained numerous examples of nonadoption and abandonment of the technology by individuals or limited success in attempts to scale up, spread, and sustain the program within and beyond the organization.

A striking finding that explained many such instances was a tendency to assume that the issues to be addressed were simple or complicated (hence knowable, predictable, and controllable) rather than *complex* (that is, inherently not knowable or predictable but dynamic and emergent). Common problems included the following:

- The technology and linked program had been designed around an oversimplified, textbook model of the condition.
- The technology was insufficiently prototyped, insufficiently customizable, insufficiently dependable, dependent on complex knowledge to use it, or designed to generate data or knowledge that was incomplete, inappropriate, or contested in the care context.
- The value proposition of the technology was unclear, in terms of a viable business venture for its developer or in terms of a clear benefit for patients and an affordable real-world service model.
- The intended users of the technology had plausible personal or professional reasons to resist or reject it.
- The organization(s) were inadequately set up for innovation, not ready for (or interested in) this particular innovation, unable to negotiate a viable business model with partner organizations, unable to shift to new ways of working, or unable to support the extensive work needed to implement and sustain the change (including the rhetorical “work” of making sense of an equivocal technology).
- Complexity in external (financial, regulatory, legal, policy) issues—of which reimbursement seemed to be particularly key—stalled the mainstreaming and spread of the program.
- The program was unable to adapt and evolve over time in a way that continued to meet the needs of its intended users and remain clinically, operationally, and financially viable.

### Strengths and Limitations

The NASSS framework has been developed systematically to fill a key gap in the literature on technology implementation—specifically, to address not just adoption but also *nonadoption* and *abandonment* of technologies and the challenges associated with moving from a local demonstration project to one that is fully mainstreamed and part of business as usual locally (scale-up), transferable to new settings (spread), and maintained long term through adaptation to context over time (sustainability).

NASSS was never intended to provide a simple fix for complex problems. It cannot be applied in a formulaic way or used deterministically as a tool, nor do we believe it is possible to make firm predictions about which elements of the framework will be mission-critical or how the different elements will interact (since these are likely to differ substantially in different cases and settings). With these caveats, we believe that, subject to further empirical testing, the NASSS diagram in Figure 2 and the NASSS domains and questions in Table 2 have several potential uses. In particular, they could be used (1) at an early stage in a technology development to inform technology and service design; (2) in strategic planning to identify technological innovations that (perhaps despite policy enthusiasm) have limited chance of achieving large-scale, sustained adoption; (3) in technology implementation projects to address the micro-level challenges of individual adoption, the meso-level challenges of organizational assimilation, and the macro-level challenges of the policy and regulatory environment; (4) to inform and support scale-up and rollout of technology programs; and (5) retrospectively to explain program failures.

The empirical work to develop NASSS was based entirely in the United Kingdom, although collaborators who peer reviewed and tested the draft framework included groups from Australia, Canada, Italy, and the United States. The 6 case studies presented in this paper have been followed for up to 3 years; field work is ongoing, so additional insights about long-term sustainability may emerge in the future. We strongly encourage other research groups to explore the applicability of the framework for different purposes and to adapt and extend it if appropriate.

### Comparison With Previous Work

Previous technology implementation frameworks identified in our hermeneutic review of the literature are summarized in Multimedia Appendix 1; the approaches and limitations of those frameworks are described in the Results section. We selected 2 rigorously developed, multilevel, integrative frameworks as most closely fitting our own empirical data [43,59] and have built on these in three main ways.

First, we have added a preliminary focus on the illness or condition for which the technology is assumed to be a solution, an emphasis that emerged from our empirical data but probably also reflects the background of several authors as clinicians, psychologists, or patient-facing service coordinators. Second, we have introduced the heuristic of classifying each domain of interest as simple, complicated, or complex, and cautiously concluded that it is *complexity in multiple domains* that poses the greatest challenge to scale-up, spread, and sustainability. Third, while acknowledging our academic audiences and not wishing to oversimplify a problem that is inherently complex, we have produced a visual representation of the different NASSS domains that we hope will be accessible to key nonacademic audiences: clinicians, managers, technology developers, executive decision makers in health and care organizations, and patients and caregivers.

### Conclusions

Implementing new technologies as part of changes to health and social care services is inherently challenging. While policy makers are calling for technology to be implemented rapidly and at scale, the reality is that when dealing with the multiple complexities of health and care, it is extremely difficult to go beyond small-scale demonstration projects. We hope that the NASSS framework will help implementation teams—and, at an earlier stage, technology and service designers—to identify, understand, and address the interacting challenges to achieving sustained adoption, local scale-up, distant spread, and long-term sustainability of their programs.

While we believe that the NASSS framework is academically defensible, additional work is now ongoing to make it accessible to its intended users beyond academia. In the United Kingdom, the NHS National Technology Adoption Centre (NTAC) was set up in 2007 to promote the uptake of health technologies through its Technology Implementation Projects and HowToWhyTo guides (which attempt to distil lessons learned from pilots in a format designed to be easily transferable and hence reusable). One of the principal objectives of the guides has been to assist practitioner stakeholders to build a business case for innovation. However, a recent study suggests that their impact has been disappointing and points to the difficulties of making context-specific knowledge transferable and hence reusable in other contexts [124]. Cognizant of this, we are currently working with design colleagues to develop and evaluate accessible infographic summaries, Web tools, and team learning opportunities based on the NASSS framework for different audiences.

## Appendix

Multimedia Appendix 1 Previous implementation/evaluation frameworks for health and care technologies.

Multimedia Appendix 2 Examples from case studies to illustrate NASSS.

## Abbreviations

ARCHIE: anchored, realistic, cocreative, human, integrated, and evaluated
COTS: customizable, off-the-shelf
GPS: global positioning system
NASSS: nonadoption, abandonment, scale-up, spread, and sustainability
SCALS: Studies in Co-creating Assisted Living Solutions
SUPPORT-HF: Seamless User-Centred Proactive Provision of Risk-Stratified Treatment for Heart Failure
VOCAL: Virtual Online Consultations: Advantages and Limitations
