# Supplementary material for: Beyond Adoption: A New Framework for Theorizing and Evaluating Nonadoption, Abandonment, and Challenges to the Scale-Up, Spread, and Sustainability of Health and Care Technologies
Source: J Med Internet Res. 2017 Nov 1;19(11):e367. doi: 10.2196/jmir.8775 (PMC5688245; doi:10.2196/jmir.8775)
Supplement: Multimedia Appendix 1 [file jmir_v19i11e367_app1.pdf]

Previous implementation/evaluation frameworks for health and care technologies

The frameworks below are specifically oriented to technology implementation. Additional frameworks for the adoption and implementation of innovations more generally are covered in the Background section of the paper.

Theory-informed integrative reviews

1. Van Gemert-Pijnen et al. [review] 2013 (diffusion of innovation, human-technology interaction, service improvement, organizational development) [43]
2. Van Dyk [review] 2014 (diffusion of innovation, e-readiness, technology acceptance and use, transactional economics, information system life cycle) [59]

Frameworks based on a logic model for developing and implementing a technology

3. Eng et al. 1999 (conceptualization/design □ implementation □ assessment/refinement) [61]
4. Hebert 2001 (structure □ process □ outcome) [62]
5. Catwell & Sheikh 2009 (inception □ requirements/analysis □ design/develop/test □ implement/deploy – each with continuous evaluation) [63]
6. Chang et al. [review] 2015 (inputs □ activities □ outputs □ outcomes, plus stakeholders) [51]

Frameworks presented as a list of criteria (or ‘things to think about’)

7. Shaw 2002 (clinical, human and organizational, educational, administrative, technical, social) [64]
8. Kazanjian & Green 2002 (population at risk, population impact, economic concerns, social context [including ethical, legal, and political concerns], technology assessment) [65]
9. Ganesh 2004 (people [patient/practitioner/provider], technical, knowledge [training], organizational, regulation/policy, social, economic) [66]
10. Dansky et al. 2006 (design and methodology, technology-related, environmental, logistic or administrative) [67]
11. Hamid & Sarmad 2008 (includes costs, benefits, ease of learning, ease of use, accessibility, compatibility, functionality, user satisfaction) [68]
12. Esser & Gossens 2009 (doctor-patient communication, technology-mediated communication, technology acceptance) [69]
13. Greenhalgh et al. 2017 (technology barriers, patient barriers, individual staff barriers, team barriers, business and financial barriers, and governance and regulatory barriers) [13]

Frameworks based on static models of systems

14. Van der Meijden et al. 2003 (mostly DeLone & McLean model for Management Information Systems based on system quality, information quality, usage attributes, user satisfaction, individual impact, organizational impact) [50]
15. Yusof et al. 2008 ‘HOT-FIT framework’ (mostly DeLone & McLean, plus leadership, communication) [49]

Framework based on individual adoption/engagement

16. O’Connor et al. 2016 (DIgital health EngaGement mOdel [DIEGO], based on normalization process theory and burden of treatment theory) [48]

Frameworks based on dynamic/developmental models of systems

17. Kushniruk 2002 (systems development life cycle) [70]
18. Kukafka et al. 2003 (extending Technology Adoption Model, social-cognitive theory, and diffusion of innovation theory with Green & Kreuter's PRECEDE-PROCEED model of complex organizational change) [58]
19. Kaufman et al. 2006 (specify needs develop components integrate components into system integrate system into clinical setting routine use of system) [71]
20. Greenhalgh and Russell 2009 (implementation and evaluation of e-health programs as contingent and political processes) [52]
21. Van Dyk & Schutte 2013 (Telemedicine Service Maturity Model) [57]
22. Abbott et al 2013 ('health IT implementation best practices', using a complex adaptive systems framing) [60]
23. Agboola 2014 ("pragmatic, multi-method, multi-phase approach" focusing on building alliances and overcoming front-line challenges) [56]
24. Greenhalgh et al. 2015: (ARCHIE principles for telehealth/telecare services: Anchored in what matters to patients; Realistic about the natural history of illness; Co-creative – evolving and adapting solutions with users; Human – supported through interpersonal relationships and social networks; Integrated, through attention to mutual awareness and knowledge sharing; Evaluated, to drive system learning) [47]
25. Mummah et al. 2016 (iterative design model, comprising empathize specify ground ideate prototype gather build pilot evaluate share) [55]
26. Wouters et al. 2016 (Technology Adoption Readiness Scale, derived from normalization process theory) [72]
27. Madjedi et al. 2016 (emphasis on partnership-building and democratic co-design with ethnic and underserved communities) [54]
28. Anton et al. 2017 (iterative design model, comprising optimise technology-enhanced services develop regulations and guidelines disseminate information improve organizational readiness provide ongoing training and technical support) [53]
