# Supplementary material for: Beyond Adoption: A New Framework for Theorizing and Evaluating Nonadoption, Abandonment, and Challenges to the Scale-Up, Spread, and Sustainability of Health and Care Technologies
Source: J Med Internet Res. 2017 Nov 1;19(11):e367. doi: 10.2196/jmir.8775 (PMC5688245; doi:10.2196/jmir.8775)
Supplement: Multimedia Appendix 2 [file jmir_v19i11e367_app2.pdf]

Examples from our case studies to illustrate Nonadoption, Abandonment and failure of Scale-up, Spread, and Sustainability

DOMAIN / QUESTION	EXAMPLES
**DOMAIN 1.**	**THE CONDITION OR ILLNESS**
1A. What is the nature of the condition or illness?	In Case A, follow-up consultations after cancer surgery and routine consultations for young adults with diabetes were fairly predictable and consistent; the few unpredictable eventualities were low-risk. In contrast, diabetes in pregnancy is a volatile condition and if poorly controlled can lead to fetal malformations or death. The physician felt strongly accountable to the unborn child so “erred on the side of caution”, inviting very few women to try the service. Similarly, heart failure was typically an unpredictable condition whose effects varied from patient to patient (and in the same patient over time).
1B. What are the relevant socio-cultural factors and comorbidities?	In the heart failure service in Case A, nurses made judgements about patients’ cognitive ability, health literacy, motivation and mental health status (notably depression), leading to many not being offered the video consultation option.<br>In the antenatal diabetes service in Case A, most patients who were invited to try video consultations (3% of the clinic population) were native English speakers, university educated and working in professional jobs (e.g. doctor, science teacher), despite the fact that most patients in this clinic were first or second generation immigrants (many with limited English) and school education only. Unless the patient had very high health literacy, IT literacy and English fluency, she was not offered the option of video consultations.
**DOMAIN 2.**	**THE TECHNOLOGY**
2A. What are the key features of the technology?	In Case A, the technologies were not 100% dependable. Heart failure nurses used the telephone to problem-solve when video connection failed (and/or during patient set-up).<br>In Case E, Product A was not available as an app (so could not be easily used on a smart phone) and lacked essential functionality for organising care such as a calendar.<br>In Case F, whilst the data warehouse for integrated case management was well embedded technically, it did not deliver the functionality required in terms of being interoperable between multiple systems, meaning that in practice significant workarounds were required to (for example) share care plans.
2B. What kind of knowledge does the technology bring into play?	In Case D, some of the staff resistance to remote biomarker monitoring was linked to a belief that such markers, however accurately measured, provided an impoverished picture of the patient’s status and that a more holistic assessment based on personal contact and conversations (“taking a proper history”) was necessary. Some patients in this study engaged enthusiastically with the data and said they had learnt a lot about their condition; others interpreted weight gain as “fat” rather than fluid overload, so were reluctant to participate in a care plan that involved repeated self-weighing.<br>In Case F, the predictive risk modelling tool generated a different kind of risk estimate than a home visit from a clinician or social worker who knew the individual well and had the capacity and authority to bear witness to a narrative and make contextual judgements; often, both were needed.

2C. What knowledge and/or support is required to use the technology?	In Case A, both staff and patients had to be 'Skype™ [or FaceTime™]-literate'. Micro-analysis of consultation transcripts showed that clinicians sometimes provided ad hoc technical support to the patient to establish and maintain a connection (e.g. connecting by telephone to talk them through set-up).<br>In Case E, both Product A and Product B required considerable knowledge and skill to use the care organizing software to its full potential, but extensive and ongoing support for users was only factored in by the latter developer (a carer support charity).
2D. What is the technology supply model?	Cases B and C both relied on generic solutions from medium sized companies, hence are potentially vulnerable to supplier withdrawal.<br>Case A also relied on generic solutions but in this case, these were mass-market software packages from large, multi-national companies, presenting low risk of market withdrawal and straightforward substitutability.<br>Case D was a prototype technology at the stage of being tested in a randomized controlled trial; there was no real-world supply model at this stage.<br>Case E featured 2 products, A and B, the former a web portal and the latter an app. Both were bespoke solutions developed by small companies, presenting high risk of market withdrawal and no obvious substitutability options.<br>Case F featured a bespoke data warehouse and user interface incorporating predictive risk modelling supplied through a longstanding relationship with a SME which was later acquired by a global company.
**DOMAIN 3.**	**THE VALUE PROPOSITION**
3A. What is the developer's business case for the technology (supply-side value)?<br>3B. What is its desirability, efficacy, safety and cost-effectiveness (demand-side value)?	Case E included two very different business models (and technology development models) for the same condition. In one (Product A), the value proposition was highly speculative and little attempt was made to work with intended end-users to increase the technology's fitness-for-purpose (and hence its desirability) during development; the implicit assumption was that the technology would be more or less plug-and-play. The business model rested on provider organizations paying for a block contract even though the intended benefits (and/or savings elsewhere in the system) were not clear. Product B included substantial up-front investment (from publicly funded R&D) to undertake co-design work; it was explicitly developed as a 'public good' in which costs to end-users would be minimal and as a *component* of a wider (and ongoing) charity-supported package. Despite obtaining R&D funding, the large amount of development work for Product B meant that an early global technology provider withdrew from the project as the value proposition – and potential for profit – diminished.
**DOMAIN 4.**	**THE ADOPTER SYSTEM (STAFF, PATIENT, LAY CARERS)**
4A. What changes in staff roles, practices and identities are implied?	In Case B, social care staff were uneasy about 'tagging' – and hence placed great emphasis on striking a pragmatic and morally justifiable balance between autonomy and safety, taking account of how client may feel about being monitored in this way.<br>In Case D, some nurses resisted remote biomarker monitoring because they were concerned that their professional practice based on 'warm care' (personal relationships with patients, reviewed through regular clinic appointments) would be largely replaced by 'cold care' (reviewing biomarkers and sending digital messages but rarely seeing the patient), though other nurses were comfortable with this change and keen to pilot the model.
4B. What input is expected of the patient (and/or immediate	In Case B, many clients with cognitive impairment did not like being tracked; some who initially accepted the device subsequently removed or sought to disable it.<br>In Case C, our index cases mostly viewed pendant alarms as helpful and easy to use,

carer) – and is this achievable by, and acceptable to, them?	but some individuals who were assessed as 'needing' an alarm refused to accept it because they did not believe they needed it, did not like the aesthetics or did not see why they should pay for it; others accepted the device but (for numerous reasons) did not wear it.
4C. What is assumed about the extended network of lay carers?	In Case B, the GPS tracking system was designed around an assumed 'hierarchy' of friends and relatives for the call center staff to telephone if the alarm was triggered, though in reality some potentially eligible clients had weak or absent social networks.<br>In Case C, the fact that the pendant alarm service in Case C could send professional staff if such a network was weak or absent may have explained its much wider uptake. One patient in Case C who was contemplating getting a pendant alarm was asked to think about nominating his next-door neighbor as a 'first response' contact. But he did not feel comfortable giving this individual what amounted to open access to his house, partly for privacy reasons but also because (he felt) this would change the nature of their neighborly relationship to one of dependency.<br>In Case E, an assumption underpinning design of the software was that a group of friends and/or relatives would not only exist and live locally but also that they would be sufficiently technology-savvy and willing to collaborate around the care of the index case. In fact, as Product B illustrated, such networks were rarely pre-existing; they often had to be built and nurtured.
**DOMAIN 5.**	**THE ORGANIZATION**
5A. What is the organization's capacity to innovate?	Some organizations in our case studies clearly lacked effective leadership and/or absorptive capacity (specific examples withheld).<br>In other cases, notably the video consulting studies in Case A, there was excellent leadership, enthusiasm for the innovation and the organization met key criteria for 'innovativeness' (e.g. it had previously won a national 'Digital Trust of the Year' award) but programs made slow progress because of lack of organizational slack (i.e. insufficient resources – see domain 6).
5B. How ready is the organization for this technology-supported change?	In some failed programs, there was no enthusiasm for the program at board level and/or key opponents were strategically placed and had high wrecking power (specific examples withheld).
5C. How easy will the adoption and funding decision be?	In Case A, the question of whether a video consultation *actually* cost less to deliver (and therefore whether it would actually make service provision more efficient) was not easy to answer because of knock-ons in the system.<br>In Case E, one organization bought a product licence for Product A but invested nothing further, assuming (wrongly) that there would be widespread uptake and efficiency savings in a short space of time.<br>In Case F, establishing 'integrated case management' as enabled through shared data and predictive risk modelling technology was extremely complex because multiple organisations needed to be involved; the establishment and development of the program unfolded over a number of years and relied on partnership working and contracting arrangements at different levels of multiple organisations. The anticipated reduction in costs from reducing hospital admissions were not realized as real savings because of the complexities of reimbursement mechanisms and because case management was not always successful in avoiding admissions.
5D. What changes will be needed in team interactions and routines?	In Case A, whilst the video consultations themselves often worked well, the linked routines for booking appointments, managing the clinic list (e.g. registering when each patient had 'arrived' and 'left') and organizing follow-up did not mesh well with a system that had evolved to process patients using their physical presence (waiting

	in line at a reception desk), physical transfer of paper records between different plastic 'bins', and sticky notes. Alignment with such routines was initially achieved using workarounds; by the end of the study, new (electronic) routines had been developed by some but not all participating teams.<br>In Case B, new protocols were devised to support the selection and deployment of GPS devices to clients, but these proved problematic and fixes to them required numerous workarounds.
5E. What work is involved in implementation and who will do it?	In Case D, wide variability in engagement among the different study sites could be explained largely in terms of the extent to which the local team shared a vision for how remote biomarker monitoring for heart failure might enhance rather than threaten the existing service.<br>In Case E, Product A, there was a substantial underestimate of implementation work from the developer, who struggled to understand and engage with local authorities, charities, practices and potential users. In contrast, the charity that co-developed Product B recognized that a great deal of preparatory work would be needed and saw this as part of its core business.
**DOMAIN 6.**	**THE WIDER SYSTEM**
6A. What is the political, economic, regulatory, professional (e.g. medicolegal) and socio-cultural context for program roll-out?	At the time of the study (most cases ran from 2015 to 2017), the UK public sector was going through a 'fiscal ice age'. Despite a strong policy discourse around technological innovation in particular, the reality at the front line was that budgets were frozen, most departments in the study were understaffed and it was often impossible to back-fill posts to allow attendance at meetings or training courses. This affected all six case studies adversely.<br>In Case A, there was no national tariff set for reimbursing a video consultation. A local workaround had been agreed between the hospital and the local commissioning organization that such consultations would be reimbursed at a rate intermediate between a telephone consultation and a face-to-face one. But even though members of the relevant national policymaking team were on the VOCAL steering group and there was no opposition "in principle" to establishing a separate tariff, a national tariff for remote consultations in the UK had still not been achieved at the time of writing.
**DOMAIN 7.**	**INTERACTION AND ADAPTATION OVER TIME**
7A. How much scope is there for adapting and co-evolving the technology and the service over time?	In Case C, a pendant alarm service initially introduced to provide *emergency physical support* (e.g. for falls) adapted over time to provide *non-emergency emotional support* for older people who were encouraged to press the alarm button when feeling lonely. Potentially remediable problems occurred with some pendant alarms in Case C but adaptation was impossible because of the risk of loss of warranty; the same problem would have occurred in Case B were it not for the researchers' ongoing dialogue with the suppliers as part of the SCALS study.
7B. How resilient is the organisation to handling critical events and adapting to unforeseen eventualities?	In Case F, following the introduction of the integrated case management data warehouse technology, clinical and administrative staff across different organizations collectively learnt and redefined what this technology could and could not do. They amended, adapted and worked around it – for example, clinicians and practitioners reviewed the outputs of the data driven risk stratification model but also supplemented these with other data and used their judgement to target patients *not* identified as 'high risk' by the model. Notwithstanding these efforts, there was a brittleness about the technology (and the work routines it required) that staff experienced as persistently frustrating.
